# Are Impulsive Decisions Always Irrational? An Experimental Investigation of Impulsive Decisions in the Domains of Gains and Losses

**DOI:** 10.3390/ijerph18168518

**Published:** 2021-08-12

**Authors:** Renata M. Heilman, Petko Kusev, Mircea Miclea, Joseph Teal, Rose Martin, Alessia Passanisi, Ugo Pace

**Affiliations:** 1 Department of Psychology, Babeş–Bolyai University, 400015 Cluj–Napoca, Romania; mirceamiclea@psychology.ro; 2 Behavioural Research Centre, Huddersfield Business School, The University of Huddersfield, Huddersfield HD1 3DH, UK; p.kusev@hud.ac.uk (P.K.); joseph.teal@hud.ac.uk (J.T.); 3 Department of People and Organisations, Surrey Business School, University of Surrey, Guildford GU2 7XH, UK; r.k.martin@surrey.ac.uk; 4 Faculty of Human and Social Sciences, UKE—Kore University of Enna, Cittadella Universitaria, 94100 Enna, Italy; alessia.passanisi@unikore.it (A.P.); ugo.pace@unikore.it (U.P.)

**Keywords:** impulsivity, decisional domain, losses, individual predictors, delay discounting

## Abstract

Intertemporal choices are very prevalent in daily life, ranging from simple, mundane decisions to highly consequential decisions. In this context, thinking about the future and making sound decisions are crucial to promoting mental and physical health, as well as a financially sustainable lifestyle. In the present study, we set out to investigate some of the possible underlying mechanisms, such as cognitive factors and emotional states, that promote future-oriented decisions. In a cross-sectional experimental study, we used a gain and a loss version of an intertemporal monetary choices task. Our main behavioural result indicated that people are substantially more impulsive over smaller and sooner monetary losses compared to equivalent gains. In addition, for both decisional domains, significant individual difference predictors emerged, indicating that intertemporal choices are sensitive to the affective and cognitive parameters. By focusing on the cognitive and emotional individual factors that influence impulsive decisions, our study could constitute a building block for successful future intervention programs targeted at mental and physical health issues, including gambling behaviour.

## 1. Introduction

Behavioural economics, an interdisciplinary field combining psychological insights into the economic theory of decision-making, addresses various relevant decisional situations [1]. Decisions involving trade-offs among costs and benefits occurring at different times are important and ubiquitous [2]. As a result, behavioural economics has devoted ample research to the topic of intertemporal choices. Intertemporal choices refer to decisions involving outcomes available at different points in time, usually between a smaller/sooner (SS) reward and a larger/later (LL) reward. Essentially, in intertemporal choices, people are required to make a trade-off between time and the value of a gain or loss. Intertemporal choices are very prevalent in daily life, ranging from simple, mundane decisions (such as choosing between going to a movie now or working on a school project that is due) to highly consequential decisions (such as choosing between eating a tasty snack daily or sticking to a healthy diet). In this context, thinking about the future and making sound decisions are crucial to promoting mental and physical health, as well as a financially sustainable lifestyle. In the present study, we set out to investigate some of the possible underlying mechanisms, such as the cognitive factors and emotional states, that promote future-oriented decisions. 

Usually, people manifest a strong preference for immediate outcomes, and future outcomes are devalued as a function of delay, a process that is referred to as delay discounting [3]. Therefore, high delay discounting rates are associated with more impulsive behaviours than lower delay discounting rates. For instance, the subjective value of €50 is greater when delivered today than when expected in a week’s time [4]. There is ample research linking maladaptive intertemporal choice patterns with societal and psychiatric problems, such as addictions [4,5,6], obesity and overeating [7], and pathological or problem gambling [8,9,10,11,12], as well as money management [2,13], financial benefits [14] and credit card debt [15]. More specifically, addictive behaviours, including pathological or problem gambling, were found to be associated with an increased level of impulsivity, thus leading to the proposal that the excessive delay discounting of future rewards may be a fundamental underlying process of addictive behaviour [16].

The fact that human decision-making is highly subjective and greatly influenced by a number of internal and external factors has been largely studied, both by economic, as well as psychology, researchers. Scholars have tried to identify the sources of variability between individuals in terms of their intertemporal preferences. A core distinction can be made between trait and state differences. Trait differences are considered to be relatively stable between individuals, and some of them have been attributed to processing differences in neural systems [17] or to genetic factors. Some studies have also confirmed that discount rates are stable for long periods of time [18]. A meta-analysis revealed that higher intelligence was associated with a lower delay discounting [19], whereas no additional delay discounting variance was explained by working memory. Overall, the results suggest that delay discounting is associated with intelligence, in part because of the processes instantiated in the anterior prefrontal cortex, a region known to support the integration of diverse information [20]. Ostaszewski [21] illustrated the role of personality variables in discounting: both extraverts and high impulsive individuals display higher discounting rates than introverts and low impulsive individuals. On the other hand, the state differences are more context-dependent and reveal changes in the decisional preferences within the same individual. The state differences reflect an individual’s ability to adapt their preferences and choices to a changing environment or their changing internal states or goals. Differences in the context or framing of decisional options have been found to alter delay discounting. In recent years, a range of contextual manipulations specific to the domain of delay discounting have been investigated and have shed light on the psychological processes involved. For instance, framing a delayed reward in terms of an acceleration arrival reduces the delay discounting, whereas framing it as a delayed outcome increases the delay discounting [22]. Other state differences that can change delay discounting are of emotional or physiological natures. A reduced delay discounting has been found after the consumption of a sugar-containing soft drink, compared with the delay discounting levels of participants who consumed a drink containing an artificial sweetener [23]. Research on emotion and cognition interactions has shown that affective states can change the subsequent choices and that emotion regulation strategies can also influence decisions [24,25]. With particular applications in intertemporal choices, there are mixed effects of induced positive or negative affective states [8]. Most experiments report an effect of an induced affect or emotional priming on delay discounting, but according to their results, both positive and negative affects/emotional priming can lead to decreased delay discounting [26]. In line with the appraisal tendency framework, it is likely that the decisional effects depend on the specific properties of the affective state; however, this speculation needs further empirical investigations. 

The large majority of studies investigating delay discounting have used methodologies that varied the amounts and delays for positive outcomes. However, considering immediate vs. delayed losses may conduce different results compared to discounting gains (possibly due to loss aversion). The concept of loss aversion arose from Prospect Theory [27,28], which describes how individuals evaluate their own outcomes in terms of potential gains and losses. People perceive losses as more unpleasant than they perceive commensurate gains as pleasant, and this effect is coined as loss aversion. In consequence, people are more inclined to prevent a loss than to obtain an equivalent gain [27]. Moreover, a general four–fold pattern has been predicted by Prospect Theory, describing risk attitudes in relation to the decisional domains and the probability of an outcome. More specifically, it is predicted that people would manifest risk-seeking preferences when there is a low probability of a gain or a high probability of a loss and risk-averse behaviours for high-probability gains or low-probability losses [27,28,29]. 

Some iconic early studies concluded that gains are discounted more than losses, and this effect has been named “the sign effect” [30,31]. Thaler [31] reported a dramatic gain–loss asymmetry, estimating that gain discount rates were three-to-ten times greater than those for losses, making people extremely more impulsive over gains than over losses. This conclusion, however, turned out to be too simplistic, according to newer research. Appelt and colleagues [32] found that people discount delayed gains (where the default is to receive a smaller gain sooner) more than accelerated gains (where the default is to receive a larger gain later). For losses, the pattern is reversed—people discount delayed losses less than accelerated losses [32]. Different intertemporal preferences also result in monetary vs. nonmonetary outcomes. Loewenstein [33] presented participants with five different positive and negative outcomes, three of which were monetary, while the remaining two were nonmonetary (i.e., a kiss from the favourite movie star and a nonlethal electric shock). The sign effect emerged for the monetary outcomes, whereas a distinct behavioural pattern resulted for the nonmonetary outcomes. For the kiss from the movie star, subjects preferred to delay the outcome for three days, presumably to savour its anticipation. For the electric shock, subjects were willing to pay substantially more to avoid a shock to be received in one or ten years than one in the immediate future. In this case, the subjects seemed to be willing to pay to avoid having to worry about the event over an extended period of time [33]. Future empirical works are needed to investigate the underlying factors that determine the discounting rates for losses and what contributes to different discounting rates for gains and losses, respectively.

In addition to behavioural investigations of delay discounting and an analysis of the possible underlying mechanisms of impulsivity, researchers have also taken a more formal investigative path. Computational models of delay discounting aim to develop mathematical functions that capture the relationship between a temporal proximity and the subjective value of an outcome [4]. Numerous investigations have indicated that people do not discount future outcomes at a constant rate. Accordingly, human decision-makers treat the near and far future very differently—they are more impulsive for goods that will arrive soon and more patient for outcomes that will arrive later [34]. This evidence suggests that the discounting function is not linear but hyperbolic. The standard hyperbolic model has dominated the field for several decades and includes a single free parameter, the discount rate *k* [35]. Higher values of the *k* parameter indicate that a person is more impulsive and has a steep discount rate of future rewards, whereas lower *k* levels reflect patience, with a preference to wait for a larger reward. Both psychologists and economists have embraced the hyperbolic discount model. As a result of the hyperbolic discount model’s popularity among scholars, various decision-making tasks were created to fit the hyperbolic discount function. The Monetary Choice Questionnaire (MCQ) [36] is a 27-item questionnaire in which participants are asked to choose between SS rewards and LL rewards. The outcomes from the MCQ can be expressed as the natural logarithm of *k* in Mazur’s [35] hyperbolic discounting model, with *k* increasing as the preference for SS rewards increases. Compared across different delay discounting methods, the MCQ emerged as providing the most precise assessment of delay discounting [9]; therefore, we selected this task to assess impulsive decisions. The success of the hyperbolic model can be partly attributed to the fact that the discount rate facilitates a direct psychological interpretation as a measure of impulsivity [4]. The theoretical insights that might emerge as a result of investigating the cognitive and emotional underlying mechanisms of an intertemporal choice could help to expand the existing computational decisional models.

Summing up, after a review of some critical papers on intertemporal choices, we argue for the necessity of addressing two major limitations currently in the delay discounting literature: (1) considering the mixed results obtained in previous studies regarding “the sign effect”, we view the investigation of the decisional domain effect (i.e., gains vs. losses) in intertemporal choices as extremely relevant and (2) an in-depth investigation of the common or specific cognitive and emotional factors that contribute to delay discounting for gains and losses.

Based on previous research, we expected to find a significant domain effect, indicating that participants show different impulsivity levels for gains compared to losses. However, due to the lack of previous research or existing mixed results on the topic and the exploratory nature of our study, more specific hypotheses regarding the individual affective and cognitive predictors of the decisional domain effect were difficult to formulate. Using a general population sample of young adults, we conducted a cross-sectional experimental study to test for the effects of the decisional domain (gain or loss) on delay discounting and investigate the potential significant affective and cognitive predictors. Considering there are significant differences between delay discounting over gains vs. losses, we argued that it would be worth extending the investigation of some of the underlying mechanisms from intertemporal choices over gains to the domain of losses. Individual differences in the current affective states and emotion regulation processes or cognitive factors such as decisional styles constitute potentially important sources of influence on intertemporal preferences. Our most important behavioural result indicated that participants apply different discount rates to future gains and losses. More specifically, participants discounted future losses to a much higher degree than future gains, showing an increased tendency towards impulsivity for losses. In addition, in both decisional domains, we found significant affective and cognitive predictors of impulsive behaviours. Our results could be a starting point for future studies designed to further unravel the individual predictors of impulsive behaviours, as well as what individual states or traits could differentiate between how people approach immediate or delayed gains and losses.

## 2. Materials and Methods

### 2.1. Participants 

One hundred and sixty-nine healthy young adults from the general population voluntarily participated in the study (*N* = 145 female). The mean age was 21.13 (SD = 4.53). All participants signed an informed consent prior to their participation, and anonymity was guaranteed for all participants. The study protocol was approved by the university’s ethics committee and followed the guidelines of the Romanian Psychologist’s Code of Conduct, as well as the guidelines of the Declaration of Helsinki.

### 2.2. Research Instruments and Procedure 

As summarised in the introduction section, a large volume of published research has focused on impulsive decisions over gains. However, significantly fewer investigations have been conducted in the domains of losses. Therefore, to address this gap, the main purpose of the study was to investigate if there are differences in impulsive decisions depending on the decisional domains of gains and losses of a monetary nature. Moreover, we set out to investigate the effects—namely, the current affective states and emotion regulation strategies and cognitive predictors, such as decisional styles—of impulsive decisions in relation to the decisional domains.

The participants were invited to a research lab to complete the online versions of the self-assessment questionnaires and a computerised version of the delay discounting decisional task. All participants were tested individually. The self-assessment questionnaires and the delay discounting task were presented in counterbalanced order. We used a cross-sectional design, with the decisional domain acting as the between-participants independent variable (*N* = 85 participants in the loss condition, and *N* = 84 participants in the gain condition). The participants were advised to respond as honestly as possible to all research instruments, as no right or wrong answers existed. Additionally, regarding the monetary choices task, the study participants were instructed to make the selections they would normally do in a real-life decisional scenario. Previous research indicated that providing monetary incentives in accordance with the selections participants made yielded similar results as in the hypothetical decision scenarios [13]. 

The Monetary Choice Questionnaire (MCQ) was used to assess the delay discounting levels [37]. The questionnaire includes a fixed set of 27 choices between smaller, immediate outcomes (smaller sooner, SS) and larger, delayed outcomes (larger later, LL). Both the amount of the immediate or delayed rewards and the waiting time, for the delayed option, varied from one selection to another. Based on the selections made in each of the 27 choice pairs, an estimate of a participant’s discount rate (*k* parameter) was computed. Kirby and colleagues [37] proposed the 27 choices, so that there are 9 levels for the *k* parameter. For example, a participant is required to select between receiving $54 today or $55 in 117 days. If the participant assesses the two options as equal, meaning the participant is indifferent between the two options, then the participant has an impulsivity level *k* = 0.00016 (the lowest level of the *k* parameter). However, if the participant chooses the immediate option, it implies a more impulsive decisional preference and, hence, a larger *k* parameter. A participant with a discount rate of *k* = 0.1 would be indifferent between receiving $15 today and $35 in 13 days. The 9 values of the *k* parameter range between 0.00016 and 0.25. A more detailed description of how to compute the delay discount rates and the complete set of monetary choices are presented in Kirby and collaborators [37] and will not be reported in this manuscript. It is important to note, however, that higher discount rates, as indexed by higher values of the *k* parameter, indicate increased impulsivity, meaning a preference towards selecting the SS options, whereas lower discount rates are indicative of more patient decisional preferences quantified by the selection of LL options.

The standard monetary choice questionnaire presents the decisional options in terms of gains. In our study, we used the procedure to assess delay discounting for positive outcomes, as presented by Kirby and colleagues [37]. In order to test for the effects of the decisional domain (i.e., gain vs. loss), we created a new version of the task in which the same monetary amounts and delay intervals were presented in terms of the expected losses. For example, similar to the gain condition, the participants were requested to make a choice between losing $54 today or losing $55 in 117 days. The same procedure was used to compute the *k* discount parameter for losses as for gains, with a higher *k* indicating an increased impulsivity in incurring a loss and a lower *k* indicating a decreased impulsivity over a loss.

The Emotion Regulation Questionnaire (ERQ) [38] is a valid instrument to assess individual differences in cognitive reappraisal (i.e., changing the way one thinks about potentially emotion-eliciting events) and expressive suppression (i.e., changing the way one behaviourally responds to emotion-eliciting events). The scale consists of 10 statements describing different modalities by which people can suppress or reappraise a current emotional state, on a 7-point Likert scale (where 1 represents a strong disagreement and 7 represents a strong agreement with the statement). Therefore, higher scores on each emotion regulation subscale indicate a more frequent use of the specific emotion regulation strategy. The psychometric properties of the original questionnaire reported by the authors [38] reflect an α = 0.79 internal consistency index for reappraisal and an α = 0.73 internal consistency index for suppression. The translated version of the instrument also has good internal consistency indexes (α = 0.74 for reappraisal and α = 0.72 for suppression) [1].

The Cognitive Emotion Regulation Questionnaire (CERQ) [39] is a multidimensional questionnaire that includes 9 cognitive and emotional coping strategies that have been previously associated with either positive or negative affective outcomes in response to stressful, threatening or traumatic life events. The 9 coping strategies assessed by CERQ are: Self-blame (thoughts of blaming yourself for what you have experienced), Acceptance (thoughts of being resigned to what has happened), Rumination (thinking all the time about the feelings and thoughts associated with the negative event), Positive refocusing (thinking of other, pleasant matters instead of the actual event), Refocus on planning (thinking about what steps to take in order to deal with the event), Positive reappraisal (thinking of attaching a positive meaning to the event in terms of personal growth), Putting into perspective (thoughts of playing down the seriousness of the event when compared to other events), Catastrophising (explicitly emphasising the terror of the experience) and Other-blame (thoughts of putting the blame for what you have experienced on others). The questionnaire is scored using a 5-point Likert scale (1 = almost never; 5 = almost always), with higher scores reflecting a more frequent use of a particular emotion regulation strategy. The alpha internal consistency indexes exceeded 0.8 for most of the 9 original coping strategies subscales. The national translation and adaptation process of the CERQ was described in Perţe and Miclea [40], showing similar internal consistency indexes as the original scale (alpha coefficients ranging between 0.75 and 0.86).

The Positive and Negative Affect Schedule–Extended questionnaire (PANAS–X) [41] was used to measure 7 specific affects: Fear, Sadness, Guilt, Hostility, Joviality, Self-Assurance and Attentiveness. The questionnaire includes 60 items that are assessed on a 5-point Likert scale, by which participants indicate the intensity of their current affective states, ranging from 1 (very slightly or not at all) to 5 (extremely). Higher scores on each affective state subscale indicate a more intense current affective state. The psychometric properties of the instrument are very good, the authors reporting an α = 0.88 internal consistency index for positive affects and an α = 0.85 internal consistency index for negative affects [41].

The General Decision-Making Style Inventory (GDMS) [42] was used to assess the cognitive individual differences that might be associated with impulsive decisions. Decision-making styles have been defined as “a habitual pattern individuals use in decision making” [43] or an individual’s characteristic manner of perceiving and responding to decision-making tasks [44]. The GDMS questionnaire assesses five distinct decisional styles, namely: rational (a thorough information search and logical evaluation of alternatives), intuitive (reliance on gut feelings and hunches), dependent (advice-seeking and reliance on others), avoidant (a tendency to escape and avoid decision situations) and spontaneous (a tendency to make fast and speedy decisions) [42]. The 25 behaviourally phrased items about making important decisions were presented to all study participants, and their agreement with each item was rated on a 5-point Likert scale (1 = strong disagreement, and 5 = strong agreement with each statement). Higher scores on each decisional style indicated a stronger preference to rely on that particular decisional style when making important decisions. The psychometric qualities of the inventory addressed in the original study [42], as well as the following research [45,46], provided compelling empirical reasons to use the questionnaire in the current study.

## 3. Results

The overall means and standard deviations are presented in Table 1 for all the dependent and predictor variables. No significant differences emerged between the predictor variables depending on the decisional condition (gain vs. loss) or in relation to the participants’ gender.

A one-factor independent measures analysis of variance was conducted with the independent variable domain of decision-making (gain or loss) and the dependent variable delay discount score (*k* parameter). The results showed that the domain of decision-making significantly influenced the participants’ delay discounting scores *F*(1, 167) = 263.02, *p* < 0.001, with a large effect size *η*^2^ = 0.61. Moreover, the results revelated that in both domains the delay discounting scores were low; however, the participants’ delay discounting scores were significantly higher in the domain of loss (*M* = 0.21, *SD* = 0.09) than in the domain of gain (*M* = 0.03, *SD* = 0.05).

Moreover, several significant correlations emerged between the predictor variables and the delay discounting parameter in both the gain and loss domains (see Table 2 and Table 3).

Furthermore, we used a multiple linear regression analysis to explore the relationships between the predictor domains of decision-making, decision style, current affective states, emotion regulation strategies and respondents’ delay discounting scores. Our results show that, in addition to the decision-making domain, rumination, decision style—avoidance and joviality bring significant contributions. The regression model was significant *F*(4, 164) = 73.07, *p* < 0.001 and accounted for 64% of the explained variance in the delay discounting scores ($R^{2}$ = 0.641). 

The further inspection of individual predictors revealed that all of the predictor domains of decision-making (*t* = −16.21, *p* < 0.001, β= −0.765), rumination (*t* = −2.13, *p* = 0.035, β = −0.100), decision style—avoidance (*t* = −2.70, *p* = 0.008, β= −0.133) and joviality (*t* = −2.22, *p* = 0.028, β= −0.111) made statistically significant contributions to the explained variance of the regression model. Importantly, the associations between the predictors and outcome variables were negative, which indicates that the lower the participants’ scores on rumination, decision style—avoidance, joviality and the decisions task in the domain of loss, the higher their scores will be in delay discounting.

## 4. Discussion

The classical results in terms of delay discounting indicate that gains are discounted more than losses, meaning that people are more impatient towards receiving a gain than they are towards incurring a loss [30,31]. Newer research has depicted a more nuanced situation, with intertemporal choices depending not only on the valence of the outcome but also on the type of commodity (monetary vs. nonmonetary). State and trait individual factors come into play and exert a strong influence on decisional preferences [8]. Considering there are significant differences between delay discounting over gains vs. losses, we argued that it would be worth extending the investigation of some of the underlying mechanisms from intertemporal choices over gains to the domain of losses. Individual differences in the current affective states and emotion regulation processes or cognitive factors such as decisional styles constitute potentially important sources of influence on intertemporal preferences. To this end, we conducted a cross-sectional experimental study to test for the effects of the decisional domain (gain or loss) on delay discounting and investigate the potential significant affective and cognitive predictors. 

Our main result confirmed that the participants applied different discount rates to future gains and losses. More specifically, participants discounted future losses to a much higher degree than future gains, showing an increased tendency towards impulsivity for losses. The behavioural pattern we observed was in conflict with the classical “sign effect” [30,31]. Nevertheless, our results are compatible with the more recent research related to both delay discounting decisions as well as other decisional contexts. For instance, Loewenstein’s research [33] illustrated different discount rates for future monetary vs. nonmonetary gains or losses. This result raised the possibility that an individual’s discount rates are not stable, but instead, they might be highly dependent on various factors pertaining to the decisional context. In fact, recent research in other decisional situations, such as risk-taking [29,47,48,49,50,51,52] or framing effects [53,54,55], has provided ample empirical support to the idea that decision preferences might be constructed and are highly sensitive to task-related and individual factors [56,57,58]. When it comes to framing effects, which refers to people’s tendency to change their risk preferences when decisional alternatives are presented in gain or loss terms, studies have shown that people are more prone to framing effects in their decisions related to human life than in situations where decisions refer to animals [53,59,60,61], money or properties [62,63], precious metal or artwork [64,65,66] or even aliens [67]. A meta-analysis [54] showed that scenarios involving human life and health lead to an increased size of the framing effect compared to scenarios relevant for business decisions, social decisions, animals or objects. In general, the results from the framing studies are compatible with the findings of other previous studies [47,54,68], drawing attention to the fact that the context of a decision is a major factor influencing the presence and even the magnitude of the framing effect. 

An extensive meta-analysis of studies relating delay discounting and addictive behaviours [9] critically discussed various paradigms of assessing delay discounting and concluded that multi-item discounting tasks provide the most precise assessment of temporal discounting. Moreover, among multiple multi-item discounting tasks that were employed in the studies reviewed in the meta-analysis, the MCQ emerged as the most adequate [9]. Green and Myerson [3] highlighted the necessity for additional research that clarified the relationship between the behavioural and psychometric measures of impulsive decisions, both in the general population as well as with specific disorders. Once again, the variations in delay discounting preferences found in some studies using different methodologies draw attention to the highly contextual nature of human decisional preferences and the fact that empirical results should be generalised with caution, as they might not apply to all decisional contexts.

In general, delay discounting paradigms associate rationality or adaptive decisions with the selection of a larger reward, in spite of the time delay to obtain said reward. Therefore, when it comes to positive outcomes, being patient and preferring the LL option is considered to be advantageous or rational. When we created the loss version of the MCQ, we used the same monetary amounts and time delays as the original gain version. In the resulting loss version, the rational or adaptive behaviour became the selection of the immediate smaller loss; thus, the impulsive choice. In conclusion, the two versions of the MCQ that we used to assess impulsivity over gains and losses captured the opposing decision preferences associated with a rational outcome.

In addition to our major goal of investigating the effects of the decisional domain on impulsive decisions, we also set out to explore potential cognitive and affective predictors. Our results indicated that both affective (such as the current emotional states or emotion regulation strategies) and cognitive (such as decisional styles) individual predictors can influence participants’ impulsive decision tendencies. These results invite further investigation, as they provide, once more, proof of the highly subjective and context dependent nature of decisional processes. The impact of emotions on impulsive decisions has been addressed in previous research (see, for instance, [69,70,71]), showing that sadness increases financial impulsivity [71], whereas gratitude has the opposite effect of reducing impulsivity [69,70]. In our study, we found that a current state of joviality is also associated with a reduced overall impulsivity in a financial decisional setting. Emotion regulation strategies have also been considered as potential predictors of impulsivity. For instance, studies [72,73] found that difficulties in emotion regulation are associated with the increased delay discounting of future gains and that the current use of expressive suppression to downregulate the affective states is linked to a decreased delay discounting of gains [74]. To the best of our knowledge, cognitive emotion regulation strategies have not been previously assessed in the context of the financial delay discounting paradigm; thus, follow-up research on this topic is warranted. Decision-making styles have been previously investigated in relation to several decisional settings or real-life behaviours, such as rationality [75], career decision-making [76,77], management decisions [78] or mental health [79]. However, there is an acute lack of research-related decisional styles for delay discounting or impulsive decisions. Our results indicated that further research should be conducted to explore the associations between the two concepts, especially considering the powerful effect of the decisional domain. More specifically, we found that an avoidant decisional style was associated with a lower impulsivity over losses, whereas a rational decisional style was correlated with less delay discounting of future gains. Nevertheless, it should be noted that the impact of the decisional domain (gains vs. losses) exerted a much higher influence on the participants’ impulsive decisions than any of the individual differences.

A common limitation of delay discounting studies, including ours, is their cross-sectional nature. Thus, whatever associations are found between delay discounting and the problematic behaviours, no directionality can be inferred. In the context of pathological or problem gambling, this means that it is not yet clear whether increased impulsivity or future discounting is a precursor for developing a gambling disorder or if it is that, once a gambling disorder has been developed, it alters one’s decisional preferences [9]. Nevertheless, an extensive review of the delay discounting studies [80] argued that delay discounting might be a trans-disease process, as impulsive decisional preferences have been found to be associated with numerous disorders and problematic behaviours. An important implication that might ensue is that the treatment for a disorder associated with maladaptive decisions may be more effective if the deficient decisional processes are targeted prior to or in conjunction with the other treatment [80].

In conclusion, incorporating psychological factors into economic models of intertemporal choice promises to increase their ecological validity and predictive power [2,81,82]. The interplay between incidental emotions effects, emotion regulation and trait-like differences, such as cognitive individual differences on intertemporal choice, presents an avenue for future research. The predictability of many of these context effects is advantageous for researchers and policymakers. Advances in the complex issue of understanding intertemporal choices can nudge people towards making more patient, better decisions [83].

## 5. Conclusions

The field of behavioural economics studies the effects of psychological, emotional, cognitive and social factors on various types of decision settings, including intertemporal choices. As a result of the mixture of theories and research methods derived from both psychology and economics, behavioural economics provides the tools to nudge people into making better choices with numerous applications for health, wealth and prosperity [83,84,85,86]. Unravelling the ways to reduce impulsive decision-making promises a cost-effective means toward better day-to-day decisions [87]. 

Our results bring important theoretical contributions to this fast-developing field by addressing some understudied key issues, such as the cognitive and affective individual differences associated with impulsive decisions over future gains and losses. As a consequence of the remarkable theoretical progress in intertemporal choices, practical applications, as well as public policies, can be promoted. Considering there are substantial intraindividual differences in impulsive decisions as a function of the contextual or situational factors, this opens the gate for studies focusing on the training and manipulations that successfully target and decrease heightened delay discounting and, in turn, address some of the maladaptive behaviours associated with impulsivity [26,88]. Overall, the results show that certain interventions can reduce delay discounting and have a lasting positive impact on the target behavioural aspect under investigation. While acceptance-based/mindfulness-based training is presented as the most promising avenue to pursue in future clinical research, a handful of studies have also investigated money management-based training under the rationale that more knowledge of money management increases the salience of future rewards [26]. The health domain has already benefitted from important applications that target delay discounting reductions in order to prevent or ameliorate physical or mental illnesses [26,89], and the fields of economics or education would also benefit from increased efforts to design strategies or public policies targeted at reducing people’s impatience levels. For instance, studies have already linked impulsive decisions with money management issues [2,13], financial benefits [14] and credit card debt [15]. By acknowledging some of the affective or cognitive factors that contribute to an impulsive decisional style, training programs or financial nudges could be applied to promote a more future-oriented perspective, such as in retirement savings behaviours. In a similar vein, in the educational field, some applications could be envisioned. For instance, programs could be designed to encourage students to focus more on the long-term benefits of education and not to succumb to short-term gains of other activities or potential immediate losses associated with allocating more time for their studies.

By focusing on the cognitive and emotional individual factors that influence impulsive decisions, our study could constitute a building block for successful future intervention programs targeted at mental and physical health issues, including gambling behaviour. 

## Figures and Tables

**Table 1:** Means and standard deviations of the study variables.

Scale	Subscale	Means	SD
Decisional styles	Rational Intuitive Dependent Avoidant Spontaneous	4.12 3.73 3.42 2.60 2.76	0.66 0.72 0.91 0.92 0.85
ERQ	Cognitive reappraisal Expressive suppression	5.04 3.37	1.05 1.45
CERQ	Self-blame Acceptance Rumination Positive refocusing Refocus on planning Positive reappraisal Putting into perspective Catastrophising Other—blame	11.73 13.73 14.98 10.46 15.57 14.70 13.56 8.98 7.82	3.38 3.16 3.62 3.99 3.04 3.86 4.40 3.31 2.52
PANAS	Fear Hostility Guilt Sadness Joviality Self-Assurance Attention	9.31 8.63 9.01 8.96 25.53 17.85 13.73	3.99 3.46 4.05 4.23 6.98 4.54 2.68

**Table 2:** Significant correlations between the cognitive and affective individual predictors and delay discounting scores (*k* parameter) in the domain of gains.

Study Variable	1	2	3
*k* parameter Decision style—Rational PANAS—Attention CERQ—Catastrophising	– −0.279 ** −0.230 * 0.236 *	– – 0.485 ** 0.351 **	– – – 0.256 *

Note: * Correlation is significant at the 0.05 level. ** Correlation is significant at the 0.01 level.

**Table 3:** Significant correlations between the cognitive and affective individual predictors and delay discounting scores (*k* parameter) in the domain of losses.

Study Variable	1	2	3	4	5
*k* parameter Decision style—Avoidant CERQ—Self-Blame CERQ—Acceptance CERQ—Rumination CERQ—Catastrophising	– −0.206 * −0.201 * −0.236 * −0.220 * −0.223 *	– – 0.290 ** −0.028 0.012 0.422 **	– – – 0.355 ** 0.489 ** 0.322 **	– – – – 0.392 ** 0.222 *	– – – – – 0.289 **

Note: * Correlation is significant at the 0.05 level. ** Correlation is significant at the 0.01 level.

## Data Availability

The data is available upon request (Renata M. Heilman—renataheilman@psychology.ro).

## References

[1] Heilman R.M. (2014). Individual differences in emotion and decision-making. Implications for economic psychology [in Romanian]. Rom. Assoc. Cogn. Sci. Publ. House ASCR.

[2] Frederick S., Loewenstein G., O’donoghue T. (2002). Time discounting and time preference: A critical review. J. Econ. Lit..

[3] Green L., Myerson J. (2004). A discounting framework for choice with delayed and probabilistic rewards. Psychol. Bull..

[4] Peters J., Büchel C. (2011). The neural mechanisms of intertemporal decision-making: Understanding variability. Trends. Cogn. Sci..

[5] Bickel W.K., Miller M.L., Yi R., Kowal B.P., Lindquist D.M., Pitcock J.A. (2007). Behavioral and neuroeconomics of drug addiction: Competing neural systems and temporal discounting processes. Drug. Alcohol. Depend..

[6] Businelle M.S., McVay M.A., Kendzor D., Copeland A. (2010). A comparison of delay discounting among smokers, substance abusers, and non-dependent controls. Drug. Alcohol. Depend..

[7] Weller R.E., Cook E.W., Avsar K.B., Cox J.E. (2008). Obese women show greater delay discounting than healthy-weight women. Appetite.

[8] Lempert K.M., Phelps E.A. (2016). The malleability of intertemporal choice. Trends. Cogn. Sci..

[9] MacKillop J., Amlung M.T., Few L.R., Ray L.A., Sweet L.H., Munafò M.R. (2011). Delayed reward discounting and addictive behavior: A meta-analysis. Psychopharmacology.

[10] Alessi S.M., Petry N.M. (2003). Pathological gambling severity is associated with impulsivity in a delay discounting procedure. Behav. Process..

[11] Petry N.M. (2001). Pathological gamblers, with and without substance use disorders, discount delayed rewards at high rates. J. Abnorm. Psychol..

[12] Petry N.M., Casarella T. (1999). Excessive discounting of delayed rewards in substance abusers with gambling problems. Drug. Alcohol. Depend..

[13] Kirby K.N., Maraković N.N. (1995). Modeling myopic decisions: Evidence for hyperbolic delay-discounting within subjects and amounts. Organ. Behav. Hum. Decis. Process..

[14] Cairns J., van der Pol M. (2008). Valuing future private and social benefits: The discounted utility model versus hyperbolic discounting models. J. Econ. Psychol..

[15] Shui H., Ausubel L.M. (2004). Time Inconsistency in the Credit Card Market.

[16] Bickel W.K., Johnson M.W., Loewenstein G., Read D., Baumeister R. (2003). Delay discounting: A fundamental behavioral process of drug dependence. Time and Decision: Economic and Psychological Perspectives on Intertemporal Choice.

[17] McClure S.M., Laibson D.I., Loewenstein G., Cohen J.D. (2004). Separate neural systems value immediate and delayed monetary rewards. Science.

[18] Anokhin A.P., Golosheykin S., Grant J.D., Heath A.C. (2011). Heritability of delay discounting in adolescence: A longitudinal twin study. Behav. Genet..

[19] Shamosh N.A., Gray J.R. (2008). Delay discounting and intelligence: A meta-analysis. Intelligence.

[20] Shamosh N.A., DeYoung C.G., Green A.E., Reis D.L., Johnson M.R., Conway A.R., Gray J.R. (2008). Individual differences in delay discounting: Relation to intelligence, working memory, and anterior prefrontal cortex. Psychol. Sci..

[21] Ostaszewski P. (1996). The relation between temperament and rate of temporal discounting. Eur. J. Personal..

[22] Weber E.U., Johnson E.J., Milch K.F., Chang H., Brodscholl J.C., Goldstein D.G. (2007). Asymmetric discounting in intertemporal choice: A query-theory account. Psychol. Sci..

[23] Wang X.T., Dvorak R.D. (2010). Sweet future: Fluctuating blood glucose levels affect future discounting. Psychol. Sci..

[24] Heilman R.M., Miu A.C., Houser D., Reuter M., Montag C. (2016). Emotion regulation and economic decision making. Neuroeconomics.

[25] Heilman R.M., Crisan L.G., Houser D., Miclea M., Miu A.C. (2010). Emotion Regulation and Decision Making under Risk and Uncertainty. Emotion.

[26] Scholten H., Scheres A., de Water E., Graf U., Granic I., Luijten M. (2019). Behavioral trainings and manipulations to reduce delay discounting: A systematic review. Psychon. Bull. Rev..

[27] Kahneman D., Tversky A. (1979). Prospect theory: An analysis of decision under risk. Econometrica.

[28] Tversky A., Kaneman D. (1992). Advances in prospect theory: Cumulative representations of uncertainty. J. Risk Uncertain..

[29] Kusev P., van Schaik P., Martin R., Hall L., Johansson P. (2020). Preference reversals during risk elicitation. J. Exp. Psychol. Gen..

[30] Benzion U., Rapoport A., Yagil J. (1989). Discount rates inferred from decisions: An experimental study. Manag. Sci..

[31] Thaler R. (1981). Some empirical evidence on dynamic inconsistency. Econ. Lett..

[32] Appelt K.C., Hardisty D.J., Weber E.U. (2011). Asymmetric discounting of gains and losses: A query theory account. J. Risk Uncertain..

[33] Loewenstein G. (1987). Anticipation and the valuation of delayed consumption. Econ. J..

[34] Rubinstein A. (2003). “Economics and psychology”? The case of hyperbolic discounting. Int. Econ. Rev..

[35] Mazur J.E., Commons M.L. (1987). An adjusting procedure for studying delayed reinforcement. The Effect of Delay and of Intervening Events on Reinforcement Value Quantitative Analyses of Behavior.

[36] Kirby K.N., Maraković N.N. (1996). Delay-discounting probabilistic rewards: Rates decrease as amounts increase. Psychon. Bull. Rev..

[37] Kirby K.N., Petry N.M., Bickel W.K. (1999). Heroin addicts have higher discount rates for delayed rewards than non-drug-using controls. J. Exp. Psychol. Gen..

[38] Gross J.J., John O.P. (2003). Individual differences in two emotion regulation processes: Implications for affect, relationships, and well-being. J. Personal. Soc. Psychol..

[39] Garnefski N., Kraaij V., Spinhoven P. (2001). Negative life events, cognitive emotion regulations and emotional problems. Personal. Individ. Differ..

[40] Perţe A., Miclea M. (2011). The standardization of the Cognitive Emotion Regulation Questionnaire (CERQ) on romanian population. Cogn. Brain Behav..

[41] Watson D., Clark L.A. (1994). PANAS-X. Manual for the Positive and Negative Affect. Schedule—Expanded Form..

[42] Scott S.G., Bruce R.A. (1995). Decision-making style: The development and assessment of a new measure. Educ. Psychol. Meas..

[43] Driver M.J., Kerr S. (1979). Individual decision making and creativity. Organizational Behavior.

[44] Harren V.A. (1979). A model of career decision making for college students. J. Vocat. Behav..

[45] Loo R. (2000). A psychometric evaluation of the General Decision-Making Style Inventory. Personal. Individ. Differ..

[46] Sadler-Smith E. (2011). The intuitive style: Relationships with local/global and verbal/visual styles, gender and superstitious reasoning. Learn. Individ. Differ..

[47] Kusev P., van Schaik P., Ayton P., Dent J., Chater N. (2009). Exaggerated risk: Prospect theory and probability weighting in risky choice. J. Exp. Psychol. Learn. Mem. Cogn..

[48] Kusev P., van Schaik P., Aldrovandi S. (2012). Preferences induced by accessibility: Evidence from priming. J. Neurosci. Psychol. Econ..

[49] Martin R., Kusev I., Cooke A., Baranova V., van Schaik P., Kusev P. (2017). Commentary: The Social Dilemma of Autonomous Vehicles. Front. Psychol..

[50] Martin R., Kusev P., van Schaik P. (2021). Autonomous vehicles: How perspective-taking accessibility alters moral judgments and consumer purchasing behavior. Cognition.

[51] Martin R., Kusev P., Teal J., Baranova V., Rigal B. (2021). Moral decision making: From bentham to veil of ignorance via perspective taking accessibility. Behav. Sci..

[52] Szasz P.L., Hofmann S.G., Heilman R.M., Curtiss J. (2016). Effect of regulating anger and sadness on decision-making. Cogn. Behav. Ther..

[53] Heilman R.M., Miclea M. (2016). Risk seeking preferences: An investigation of framing effects across decisional domains. Cogn. Brain Behav..

[54] Kühberger A. (1998). The Influence of Framing on Risky Decisions: A Meta-analysis. Organ. Behav. Hum. Decis. Process..

[55] Levin I.P., Schneider S.L., Gaeth G.J. (1998). All Frames Are Not Created Equal: A Typology and Critical Analysis of Framing Effects. Organ. Behav. Hum. Decis. Process..

[56] Heilman R.M., Kusev P. (2020). Personal Values Associated with Prosocial Decisions. Behav. Sci..

[57] Hill T., Kusev P., van Schaik P. (2019). Choice under risk: How occupation influences preferences. Front. Psychol..

[58] Kusev P., Purser H., Heilman R., Cooke A.J., van Schaik P., Baranova V., Martin R., Ayton P. (2017). Understanding risky behavior: The influence of cognitive, emotional and hormonal factors on decision-making under risk. Front. Psychol..

[59] Bloomfield A.N. (2006). Group size and the framing effect: Threats to human beings and animals. Mem. Cognit..

[60] Peters E., Levin I.P. (2008). Dissecting the risky-choice framing effect: Numeracy as an individual-difference factor in weighting risky and riskless options. Judgm. Decis. Mak..

[61] Schneider S.L. (1992). Framing and conflict: Aspiration level contingency, the status quo, and current theories of risky choice. J. Exp. Psychol. Learn. Mem. Cogn..

[62] Fagley N.S., Miller P.M. (1997). Framing effects and arenas of choice: Your money or your life?. Organ. Behav. Hum. Decis. Process..

[63] Kühberger A., Schulte-Mecklenbeck M., Perner J. (1999). The Effects of Framing, Reflection, Probability, and Payoff on Risk Preference in Choice Tasks. Organ. Behav. Hum. Decis. Process..

[64] Jou J., Shanteau J., Harris R.J. (1996). An information processing view of framing effects: The role of causal schemas in decision making. Mem. Cognit..

[65] Wang X.T. (1996). Domain-specific rationality in human choices: Violations of utility axioms and social contexts. Cognition.

[66] Wang X.T. (1996). Framing effects: Dynamics and task domains. Organ. Behav. Hum. Decis. Process..

[67] Wang X.T., Simons F., Bredart S. (2001). Social cues and verbal framing in risky choice. J. Behav. Decis. Mak..

[68] Mandel D.R., Vartanian O., Vartanian O., Mandel D.R. (2010). Frames, Brains and Content Domains: Neural and Behavioral Effects of Descriptive Context on Preferential Choice. Neuroscience of Decision Making.

[69] DeSteno D., Li Y., Dickens L., Lerner J.S. (2014). Gratitude: A tool for reducing economic impatience. Psychol. Sci..

[70] Dickens L., DeSteno D. (2016). The grateful are patient: Heightened daily gratitude is associated with attenuated temporal discounting. Emotion.

[71] Lerner J.S., Li Y., Weber E.U. (2013). The financial costs of sadness. Psychol. Sci..

[72] Malesza M. (2019). Relationship between emotion regulation, negative affect, gender and delay discounting. Curr. Psychol..

[73] Malesza M. (2019). Stress and delay discounting: The mediating role of difficulties in emotion regulation. Personal. Individ. Differ..

[74] Lawyer S.R., Jenks C.W. (2020). Emotion suppression decreases delay discounting for monetary outcomes. Psychol. Rec..

[75] Curşeu P.L., Schruijer S.G. (2012). Decision styles and rationality: An analysis of the predictive validity of the General Decision-Making Style Inventory. Educ. Psychol. Meas..

[76] Galotti K.M., Ciner E., Altenbaumer H.E., Geerts H.J., Rupp A., Woulfe J. (2006). Decision-making styles in a real-life decision: Choosing a college major. Personal. Individ. Differ..

[77] Singh R., Greenhaus J.H. (2004). The relation between career decision-making strategies and person–job fit: A study of job changers. J. Vocat. Behav..

[78] Sadler-Smith E. (2004). Cognitive style and the management of small and medium-sized enterprises. Organ. Stud..

[79] Bavolar J., Orosová O.G. (2015). Decision-making styles and their associations with decision-making competencies and mental health. Judgm. Decis. Mak..

[80] Koffarnus M.N., Jarmolowicz D.P., Mueller E.T., Bickel W.K. (2013). Changing delay discounting in the light of the competing neurobehavioral decision systems theory: A review. J. Exp. Anal. Behav..

[81] Loewenstein G., Prelec D. (1992). Anomalies in intertemporal choice: Evidence and an interpretation. Q. J. Econ..

[82] Loewenstein G., Thaler R.H. (1989). Anomalies: Intertemporal choice. J. Econ. Perspect..

[83] Thaler R.H., Sunstein C.R. (2003). Libertarian paternalism. Am. Econ. Rev..

[84] Heilman R.M., Kusev P. (2017). The gender pay gap: Can behavioral economics provide useful insights?. Front. Psychol..

[85] Thaler R., Sunstein C. (2008). Nudge: Improving Decisions about Health, Wealth, and Happiness.

[86] Kusev P., van Schaik P., Tsaneva-Atanasova K., Juliusson A., Chater N. (2018). Adaptive anchoring model: How static and dynamic presentations of time series influence judgments and predictions. Cogn. Sci..

[87] Puaschunder J.M. (2017). Nudgitize Me! A Behavioral Finance Approach to Minimize Losses and Maximize Profits from Heuristics and Biases. Int. J. Manag. Excel..

[88] Rung J.M., Madden G.J. (2018). Experimental reductions of delay discounting and impulsive choice: A systematic review and meta-analysis. J. Exp. Psychol. Gen..

[89] Shevorykin A., Pittman J.C., Bickel W.K., O’Connor R.J., Malhotra R., Prashad N., Sheffer C.E. (2019). Primed for Health: Future Thinking Priming Decreases Delay Discounting. Health Behav. Policy Rev..

